# Panama Papers' offshoring network behavior

**DOI:** 10.1016/j.heliyon.2020.e04293

**Published:** 2020-06-26

**Authors:** David Dominguez, Odette Pantoja, Pablo Pico, Miguel Mateos, María del Mar Alonso-Almeida, Mario González

**Affiliations:** a Escuela Politécnica Superior, Universidad Autónoma de Madrid, 28049 Madrid, Spain; b SIGTI-Research Group, Escuela Politécnica Nacional, Quito, Ecuador; c SI2-Lab, FICA, Universidad de las Américas, Quito, Ecuador; d FCEE, Universidad Autónoma de Madrid, 28049 Madrid, Spain

**Keywords:** Panama Papers, Offshore societies, Tax havens, Geographical networks, Graph Theory, Network Analysis, International Relations, Money, Globalization, Business, Economics

## Abstract

The present study analyzes the offshoring network constructed from the information contained in the Panama Papers, characterizing worldwide regions and countries as well as their intra- and inter-relationships. The Panama Papers 2016 divulgence is the largest leak of offshoring and tax avoidance documentation. The document leak, with a volume content of approximately 2.6 terabytes, involves more than two hundred thousand enterprises in more than two hundred countries. From this information, the offshore connections of individuals and companies are constructed and aggregated using their countries of origin. The top offshore financial regions and countries of the network are identified, and their intra- and inter-relationship are mapped and described. We are able to identify the top countries in the offshoring network and characterize their connectivity structure, discovering the more prominent actors in the worldwide offshoring scenario and their range of influence.

## 1 Introduction

The *Panama Papers* refer to a recent leak of 11.5 million of documents, uncovered by the International Consortium of Investigative Journalists (ICIJ), of confidential financial and legal documents from the Panamanian law firm “Mosack Fonseca” which offered corporate services and became one of the largest entities to provide offshore financial services worldwide (Joaristi et al., 2019). These papers provide information on the financial details of individuals and public officials, exposing the use of offshore business for possible illicit activities. Approximately 360,000 businesses and individuals are involved in this filtration, covering approximately 200 countries, which are connected to offshore structures. Behind this fraudulent scandal, illegal operations of money laundering, tax evasions and fraud have been detected. The initial purpose of tax havens was to provide low tax provisions to attract investments (Dharmapala and Hines, 2009b; Bucovetsky, 2014), but the increase of offshoring activities has become more visible, and thus, not all tax havens are perceived to be safe (Dharmapala and Hines, 2009b), with an existing tendency to use tax havens for criminal purposes.

Tax regulatory entities and authorities worldwide have focused their attention on implementing schemes to recover tax on offshore investments (Gould and Rablen, 2020). This is reinforced by the fact that 10% of world GDP is retained in tax havens, for example, countries such as the United States, have estimated annual loss of tax collection of about $30–40 billion, due to offshore activities (Gould and Rablen, 2020). Corporations are identified as the main tax haven users (O'Donovan et al., 2019), but wealthy individuals also evades taxes through the creation of a shell company (Joaristi et al., 2018). The Panama Papers showed how wealth can be hidden and taxes evaded through tax havens (Ait Bihi Ouali, 2020), however, there is a lack of transparency regarding the offshore network, which leads to the need to study its structure and behavior.

Taking into account the actual necessity to illustrate the behavior of worldwide offshoring activities, the present research aims to analyze a network of connections between countries and regions involved in this practice. The network is built based on the relations that appear in the ICIJ Offshoring and Panama Papers database (Offshore Leaks Database, 2018) and allows for the detection of association patterns between actors. The network is represented in the scale of countries and geographical regions, allowing researchers to discover the structure of the worldwide offshoring connections for the top countries in terms of occurrences in the ICIJ database. The intra- and inter-relationships between these top actors are described, and their connectivity structure is characterized.

Most previous research has focused on developed countries (Desai et al., 2004; Gumpert et al., 2016) with little research on developing countries (Buckley et al., 2013). The present research analyzes global tax haven behavior and the relationships among world-wide countries and regions. Therefore, the main contributions of this paper, are, first, to shed light on geography of money and finance and, second, to illuminate international offshoring networks. Thus, new insight into the role they play in the global economy can be stressed.

This work importance can be highlighted on its use as an input for the development of tax planning and financial control mechanism. Suspicious countries can be detected in the offshore leaks network presented in this paper, making possible to uncover the underlying structures of these networks allowing a more transparent actions regarding tax evasion, corruption, and money laundering. The research of the structure and organization of existing offshoring might benefit, on the one hand, to academics and scholars and to increasing the literature on tax haven networks. On the other hand, can help regulatory agencies and policy makers to regulate tax evasion reducing capital flight.

The document is organized as follows. In Section 2: Literature Review, the principal elements of the Panama Papers and illegal offshoring operations are detailed. In Section 3: Methodology, we describe how the data from the offshore-leak database was processed and organized, and how the offshoring network was constructed from the data. In Section 4: Results, we present the main outcomes of characterizing the intra- and inter-relationships for the top geographical regions and top countries in the offshoring network. Finally, Section 5: Conclusions, wraps up the paper and discusses the implications of our findings.

## 2 Literature review: worldwide offshoring activity

After the Panama Papers revelation, offshoring companies established in tax havens, have been in the focus of the public interest, constituting a crucial issue to be considered. Tax-havens regions with low tax rates, promoted with the objective of enhancing foreign investment, have increased in the last 25 years (Dharmapala and Hines, 2009a). At this point, Mossack Fonseca played an important role in helping companies and individuals establish operations in offshore jurisdictions, with the aim to avoid the detection by tax authorities, thereby gaining anonymity and secrecy regarding their operations.

The offshore financial economy is a corruption alternative that reinforces capital flight and tax evasion because the offshoring entities owners have the “benefit” of not reporting their profits and incomes to their respective tax authorities. Considering the veil of secrecy that offers information protection, the financial operations and ownership are completely obscured, allowing natural persons and corporations that are non-residents in tax havens to accumulate financial capital (Christensen, 2011). Tax havens, among their supposed benefits, include the banking and commercial information protection. In this way, a blindness exists regarding company owners' identities that designates second persons who act as nominated directors, thereby hiding the real owners and offering the privilege of creating accounts that are not registered publicly (Jalan and Vaidyanathan, 2017).

The principal individuals related to offshoring entities are, corrupt officials, sportspeople, politicians, movie stars and drug dealers, all of them interested in hiding their assets in tax havens. Considering that these individuals want to hide theirs incomes from illegal transactions or to evade income tax, all of them use tax havens as an illegal alternative (Stryker, 1998; Hebous and Lipatov, 2014). In the case of corporations, tax evasion is frequently used to hide illegal activities, such as “market rigging, insider trading, illicit political donations, embezzlement, fraud, and payment of bribes and commission kickbacks” (Christensen, 2011, p. 178).

One of the characteristics of tax havens is that they are small countries, with a population below 1 million people; approximately 15% of countries are tax havens (Dharmapala and Hines, 2009a). According to information collected from Fortune 500 in 2014, 358 companies (71.6%) had a minimum of 7,622 tax-haven subsidiaries (Akamah et al., 2016). This figure represents, an evasion of approximately $90 billion USD in federal taxes. There are approximately 50-60 tax havens, where more than the 30% of the global foreign direct investment is located (Haberly and Wójcik, 2014). There is a tendency for services companies to use offshore activities to a lesser extent than do manufacturing industries (Gonzalez-Navarro and Quintana-Domeque, 2016). In the same way, private firms have a greater tax reduction thanks to tax havens than public companies do (Aziz and Thornton, 2015).

The practice of offshore dissemination has multiple implications; for example, the tax base of non-haven countries deteriorates since the amount collected from taxes decreases (Bovi and Cerqueti, 2014; Jalan and Vaidyanathan, 2017). Likewise, tax havens promote the demoralization of honest companies, when they pay their tax and compare their expenditures with other companies who did not, thereby losing the faith in the system. Another consequence of tax evasion is the additional controls that countries must apply to pursue and stop this practice, increasing regulation costs.

At a global level, there is a repudiation for these practices that hide illegal acts; existing groups criticize and combat these criminal practices, and such groups are composed of “politicians, regulators, citizen groups, and the media related to tax-haven operations”, which are aware that the objective of such entities is to avoid the payment of tax in their countries (Akamah et al., 2016, p. 2). In this sense, we build the Offshoring network to map the connectivity and interactions among countries and geographical areas worldwide to determine the top actors of offshoring financial operations in the ICIJ database and their range of influence.

## 3 Methodology

In this section, the process implemented to build the offshoring networks is detailed. The first step is to organize the data from the ICIJ offshore database using PostgreSQL to extract the relevant information to identify the connections between actors by querying the database. Once the information is extracted, the network is modeled, weighting pairs of countries' connectivity by aggregating the different occurrences in the database.

### 3.1 Data collection and organization

The data were obtained from the ICIJ Panama Papers Offshore Leaks Database (https://offshoreleaks.icij.org/), where the data can be accessed and downloaded. The data has information of entities, intermediaries, officers, addresses and edges between them. A PostgreSQL database was built from the aforementioned available data, to facilitate the extraction of information through database queries.

The “ICIJ Offshore” database consists of a set of relationships between companies and individuals with offshore companies based in tax havens. The structure of these relationships are listed in: (i) Entities, (ii) Intermediaries, (iii) Officers, (iv) Addresses, (v) Edges. The aforementioned relationships are accessible from the ICIJ database as csv files and are detailed in Table 1.

**Table 1:** ICIJ database.

Table (csv file)	Content (attributes)
**Entities** correspond to enterprises created in tax havens and includes general information and commercial registration. There are 495,309 entities with the following attributes:	name, original_name, former_name, jurisdiction, jurisdiction_description, company_type, address, internal_id, incorporation_date, inactivation_date, struck_off_date, dorm_date, status, service_provider, ibcRUC, country_codes, countries, note, valid_until, node_id, sourceID.

**Intermediaries** correspond to law firms or other intermediaries that offer offshoring services: There are 24,183 intermediaries with the following attributes:	name, internal_id, address, valid_until, country_codes, countries, status, node_id, sourceID, note.

**Officers** correspond to individuals or companies with a role in the offshore entity. There are 370,873 officers with the following attributes:	name, icij_id, valid_until, country_codes, countries, node_id, sourceID, note.

**Addresses** corresponds to postal addresses of companies and individuals in the database. There are 151,665 addresses and the attributes are:	address, icij_id, valid_until, country_codes, countries, node_id, sourceID, note.

The **Edges** data contains the existing relations between all the above tables and is the product of text mining the Panama Papers. The attributes available are:	node_1, rel_type, node_2, sourceID, valid_until, start_date, end_date. Here node_1 refers to the issuing entity and node_2 refers to the receiver entity.

We use the **Edges** relationship (rel_type) information to build the connectivity between node_1 and node_2. There are 19 types of relationships, such as shareholder, beneficiary of, intermediary, president, secretary, director, to mention only a few. Relating these relationships together with the emitting and receiving nodes, the weight and direction of the network connections are built. All this information is tracked to the country of origin of individuals and companies, which are indexed by the field sourceID present in all csv files.

### 3.2 Network construction

To build the network, a set of database queries are performed to relate pairs of countries. The weight connection between pairs of countries is proportional to the number of occurrences from the queries obtained for all possible relations according to **Entities, Intermediaries, Officers, Addresses and Edges**. The resulting connectivity from a query is a directed link, using the information node_1 and node_2 from **Edges**. Inner join subqueries are performed to acquire all possible relations between each pair of countries. Finally, a high level query is performed to aggregate all the results of similar tuples returned in the subqueries. In this way, one obtains a raw value for the total number of occurrences $a_{ij}^{r}$ of pairs of countries i,j for each type of relationship *r*. These values are normalized between 0 and 4 for each of type of the 19 types of relationships r∈{0,1,…,19} and for each country *i*. The normalization $T_{r}$ and the weight of the relationship $R_{ij}$ between countries *i* and *j* is calculated as follows:

(1) $$R_{ij}=\sum_{r}T_{r},\;T_{r}=\frac{a_{ij}^{r}}{max(a^{r})max(a_{i})}\times 4,\;r\in \{0,1,\ldots ,19\}.$$

Then, the network connectivity matrix $C_{ij}$ is built as follows:

(2) $$C_{ij}=\left\{ \begin{array}{c} 1,\text{ if }R_{ij}>\theta ,\\ 0,\text{ otherwise}. \end{array}\right.$$

Here, *θ* is a trimming threshold value and it is related to the value of the desired cut-off above, of which the weight of the relationship will be considered as a connection between two countries. One can also use a quantile to obtain a value of *θ* to build the chord diagrams obtained in the next section, Section 4. In the latter case, the relations $R_{ij}>\theta_{q}$, where $\theta_{q}$ relates to the *q*-th quantile, will be used as the trimming level for the connection weights.

Then, the offshoring network is described by the adjacency matrix $O_{ij}=C_{ij}R_{ij}$. This adjacency matrix ***O*** is used to characterize the offshoring network structure between countries using chord diagrams. As mentioned, this adjacency matrix corresponds to a directed network, thus forming a non-symmetric matrix. Self-connectivity is allowed, since node_1 and node_2 from **Edges** could correspond to an individual or company within the same country.

### 3.3 Network characterization

The network structure obtained in the previous subsection is characterized for countries and mesoscopic geographical relations. The top countries in the database are identified according to their number of occurrences, and a cut-off is identified for the top 22 countries, whose occurrence frequencies are modeled by a power function.

Once the top countries are identified, a chord diagram is used to depict the intra- and inter-relationships between the blocks of the mesoscopic network, in the case of geographical regions, and between the top countries (see Section 4). The blocks (regions or countries) are arranged radially around a circle, and the relationships are drawn as arcs that connect the blocks. A connection is represented as internal when it connects a country itself or, in the case of geographical regions, when it connects countries belonging to the same regions. Chord diagrams are a highly intuitive way to depict the structure of networks and have been used to describe migration flows (Abel and Sander, 2014), enterprise sustainability reporting (González et al., 2015, González et al., 2017), and offshoring maps (Dominguez et al., 2018) to mention only a few.

To construct the chord diagrams that are presented in Fig. 6 and Fig. 3, the strength of these internal and external connections is measured. To determine the size of the connection, the chord diagram considers the number of links between enterprises among the regions/countries and the size (weighted degree) of the region/country where the connections originated. The gaps between the connections and the blocks indicate the link direction, as detailed in the next section.

## 4 Results: offshoring network structure and behavior

This section introduces the overall behavior of occurrences for countries in the offshore leak database using a histogram and modeling the occurrence frequencies. The offshoring network obtained, as explained in the previous Section 3, is presented, and the network structure is discussed in terms of countries and their geographical representation. The connectivity structure of the top countries in the database is described. Also, the results are summarized for the world geographical regions, analyzing their inter- and intra-relationships.

### 4.1 Countries' occurrences behavior in the Offshore Leaks database

**Figure 1:**
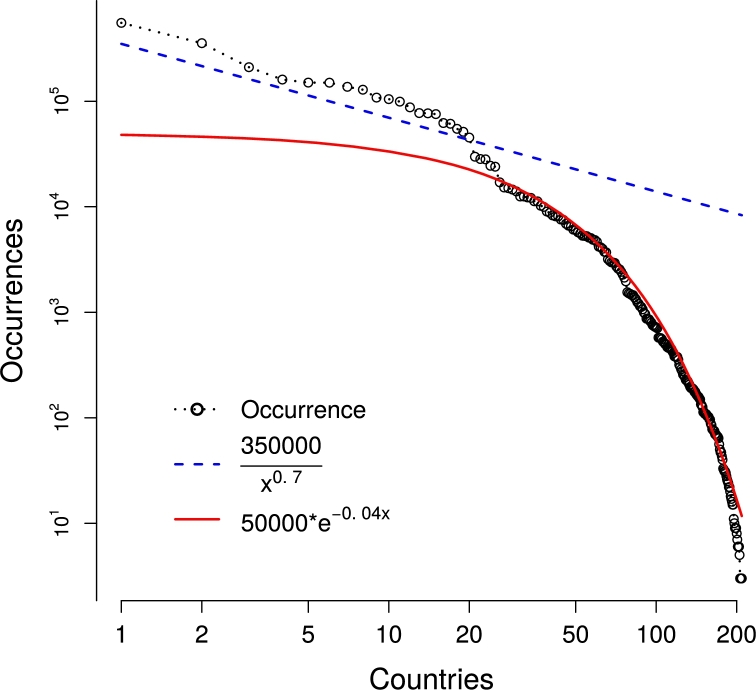
Countries histogram ordered by number of occurrences in the Offshore Leaks database, represented with black circle markers. Power law distribution for top countries *P*(*x*)∝*a*⁎*x*^(−*c*)^ in blue dashed line. Exponential distribution for the rest of countries *P*(*x*)∝*a*⁎*exp*(−*c*⁎*x*) in red solid line.

Fig. 1 depicts the behavior of the counties' occurrences in the Offshore Leaks database. The figure plots the number of Offshores per countries, ranked from the largest occurrence to the smallest. The *X*-axis represents the list of countries in decreasing order according to their occurrences in the database, which is represented in the *Y*-axis. The corresponding graphic can be seen as an histogram for the Offshore frequencies. Both axes are in logarithmic scale. Approximately the first twenty countries (larger occurrences) are fitted to a power function, while the second half is fitted to an exponential function (less frequent). These top countries' occurrences decay regularly and likely have a similar behavior independent of the scale of the number of occurrences. These are the larger tax havens identified, and their interrelationships are described in the rest of the analysis. The rest of the countries decay in such a way that the frequency variation rate is faster. These are the countries with a marginal number of connections in the network and that will eventually be disconnected by the effect of the threshold parameter used to build the networks.

Let us take a closer look at each region of the histogram. The first countries are adjusted by a power-law distribution, $P(x)\propto a⁎x^{\left(-c\right)}$, which looks lineal in a log-log scale. It fits the left part of the histogram well, for large frequencies, with a power of c=0.7, which falls between y=2/3 and y=3/4. Considering it is a probability distribution for the Offshores, c<1 implies a heavy tail, which yields to a divergence if it was not bounded for a given finite critical rank $X_{c}$.

The power-law is also known as a Pareto distribution (Schumpeter, 1949), in the economics field, or a scale-free distribution (Barabási and Albert, 1999), in numerous of other fields, such as language, history, geography, biology or physics. For instance, it can model a variety of physical or social phenomena, such as the sizes of avalanches of snow or rocks in a mountain, sand piles, earthquake magnitudes, firm sizes, city sizes, income and wealth, stock market activities, international trade, or neural spike delay, all of which starts from a simple hypothesis about the neighborhood of the interaction process (Marković and Gros, 2014).

It was also found that the presence of hubs will give the degree distribution a long tail, indicating the presence of nodes with a much higher degree than most other nodes. Among the explanations, the theory for random graphs predicts that the number of connections of an evolutionary network will be described by power-law, with c=a/b, where *a* is the born tax for new connections, and *b* is growth tax of the previous connections (Gabaix, 2009). According to this, and taking into account the fit of the Offshore power-law distribution, one may conjecture that the born tax of new Off-shores are a=2−3, while the growth tax of old Offshores is b=3−4.

It is worth mentioning that all of these scale-free phenomena are based in a competition between external drivers and inner dynamics. The inner forces are dissipative, being guided by an optimization principle. One may speculate that the role of the dissipation in the Offshores is played by optimizing their owner profits, which is equivalent to minimizing their loss, both from legal taxes of their original countries, or from the cost of hiding capital in the tax-haven jurisdiction.

Finally, the remaining countries in the region of low frequencies fit an exponential distribution P(x)∝a⁎exp(−c⁎x), describing the events in a Poisson process, in which events occur continuously and independently at a constant average rate. Such a distribution does not require an upper cutoff because it is always convergent. The meaning of the value c=0.04 found in our fit is related to the time scale of the process, and since we have not considered either the time of each Offshore in our database, or compared our results to other databases from a different time, nothing can be concluded with respect to *c*. However, it might be worth considering new releases of the Offshore documents, to consider the evolution of *c*.

### 4.2 Offshore map relations for countries

**Figure 2:**
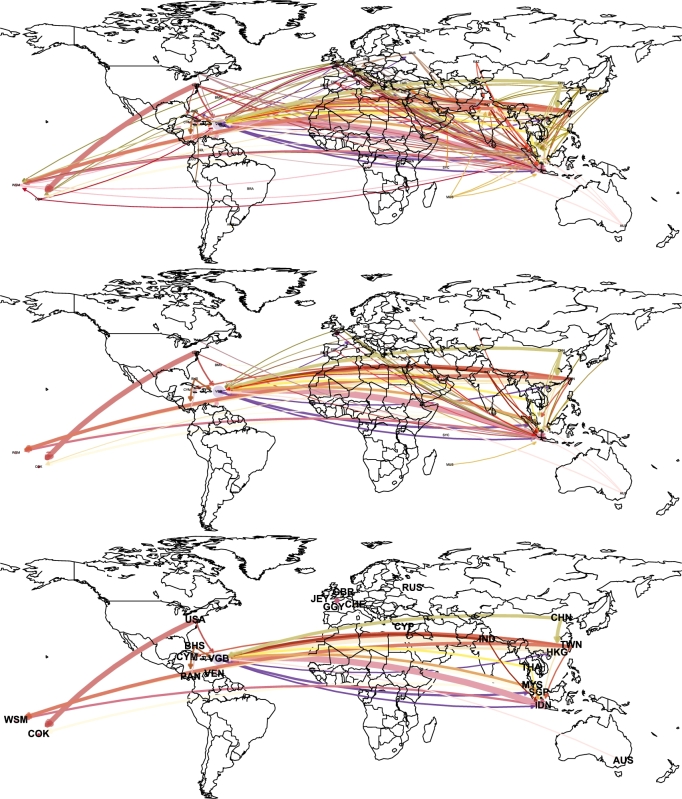
Geographic representation of offshoring countries and their relationships. From top to bottom, edge are trimmed for weights below thresholds: *θ* = {0.5,1.5,2.5}.

In Fig. 2, a geographic representation of the principal countries in the offshoring network is depicted. The countries are connected in a directed way, that is, the outgoing and incoming connection may differ. Note that both directions can occur if they exceed the indicated threshold. The countries can also have self connections, accounting for mainly internal operations. From top to bottom (in Fig. 2) the thresholds values of θ={0.5,1.5,2.5} are used. For θ=0.5, a small level of trimming is performed, thus the top panel depicts a very connected world-wide offshoring network. For θ=1.5 in the middle panel, Europe countries connections start to dilute. The behavior for Europe which becomes disconnected when increasing the trimming threshold, indicates that enterprises from that regions are more lawful players in terms of taxing and financial fair play (Gould and Rablen, 2020). Finally, the extensive trimming (high value of θ=2.5) of the offshoring network allows to discover the top countries in the offshoring network which is in correspondence with the histogram in Fig. 1.

**Figure 3:**
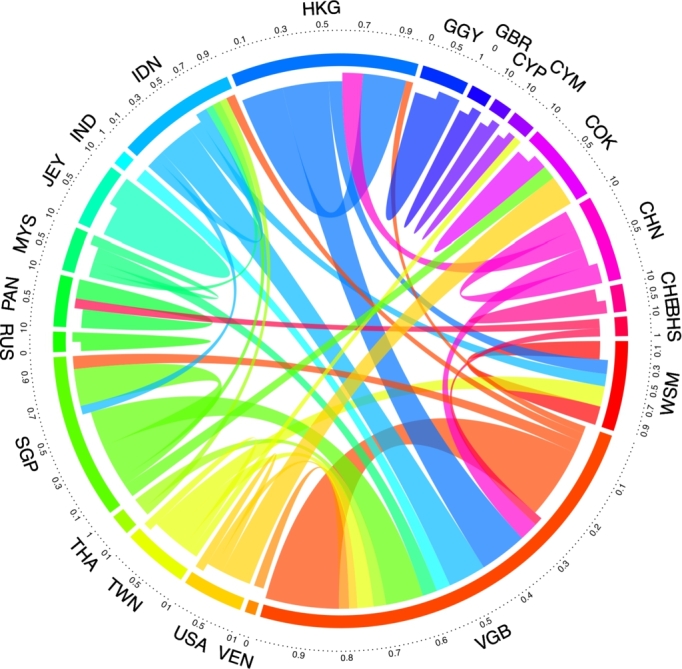
Offshoring map (chord-diagram) for top countries. Top 0.1 percent of connections between countries. Quantile *q* = 0.999, trimming for weights below threshold of *θ*_*q*_ = 2.92. See acronyms of countries in Table 2.

The relationships between the top countries are discussed in detail using the chord diagram depicted Fig. 3. The top countries involved are the Bahamas, the British Virgin Islands, the Cayman Islands, China, the Cook Islands, Cyprus, Guernsey, Hong Kong, India, Indonesia, Jersey, Malaysia, Panama, Russia, Samoa, Singapore, Switzerland, Taiwan, Thailand, the United Kingdom, the United States and Venezuela (see Table 2).

**Table 2:** Top Countries' acronyms per ISO-3166 standard.

Country	Acronym
Bahamas	BHS
British Virgin Islands	VGB
Cayman Islands	CYM
China	CHN
Cook Islands	COK
Cyprus	CYP
Guernsey	GGY
Hong Kong	HKG
India	IND
Indonesia	IDN
Jersey	JEY
Malaysia	MYS
Panama	PAN
Russia	RUS
Samoa	WSM
Singapore	SGP
Switzerland	CHE
Taiwan	TWN
Thailand	THA
United Kingdom	GBR
United States	USA
Venezuela	VEN

Fig. 3 shows the offshoring relationships for the top 0.1 percent of connections in the network. The countries (nodes) that remained connected after the trimming are the ones shown in the chord diagram. The British Virgin Islands (VGB) occupies the greater proportion in the representation of the offshoring map, and it is recognized by Mossack Fonseca's in the Offshore Leaks Database files as the favorite tax haven, where approximately 113 thousand entities are registered, 1819 of which are linked with Taiwan. Likewise, the VGB have an important volume of operations with countries such as Singapore, Malaysia, India, Indonesia, Hong Kong and China.

Hong Kong (HKG) is considered as another trendy tax haven in the Panama Papers when companies are looking for tax avoidance/evasion (Bryane and Goo, 2016). Approximately 90% of Hong Kong's gross domestic product (GDP) comes from the service industries, with the financial services included as one of the five most important industry services. The principal relations detected are with the British Virgin Islands, with Samoa to a lesser extent and with companies and persons from the same country. It has been found that such origins of evaded tax are related to money laundering and financing of terrorist activities, with approximately 500 entities connected with Hong Kong (Wang, 2017).

Singapore (SGP), despite having a business-friendly tax regime, maintains a higher connection with the British Virgin Islands, as was previously mentioned, and with the Cook Islands (COK), both of which are no-tax territories. There are 4027 registered entities in VGB related to Singapore and 48 registered in COK. Similarly, Singapore has reported 706 offshore own entities related to itself by operations that are supported by flexible tax politics.

Samoa has been an offshore finance center since 1987 and is the sixth most popular tax havens in the Panama Papers; it is focused on provisions for international business companies, since their principal market comprises countries from South East Asia (Rawlings et al., 2005). In the Offshore Leaks Database, it is possible to confirm the existence of 265 offshore entities in Samoa's jurisdiction linked to Taiwan (Offshore Leaks Database, 2018).

China (CHN) has links with Hong Kong, being their first investor, followed by the British Virgin Islands (Weichenrieder and Xu, 2015). China, like India, has propitiated zones with special economic regulations with attractive tax systems to attract offshoring investors. Taiwan's (TWN) offshore entities establish relations predominantly with Samoa (WSM) and the British Virgin Islands (VGB).

The Cook Islands (COK) are a group of Pacific islands that “concentrates on forming trusts to protect assets from seizure by courts, wives, husbands or creditors” (Vlcek, 2013, p. 652). Their principal relations are with companies from the same country, though they are also linked as an operations receiver from Singapore and the United States (USA); the connection with USA is the larger of the two. It is worth noting that the USA maintains its largest volume of offshore operations with the Cook Islands and, to a lesser extent, with the British Virgin Islands.

Panama (PAN) is known as a popular tax haven, having predominantly internal relationships and, to a minor scale, a relationship with the Bahamas (BHS), with 1120 offshore entities with jurisdiction in BHS linked with PAN. Malaysia (MYS), although having a lower weight compared to the other countries analyzed, has principal operations with the British Virgin Islands and Indonesia (IDN). Jersey (JEY) is another hosts of offshore finance centers; the operations from this activity represent 90% of its government revenues (Hampton and Christensen, 2002). Their principal operations are related to other entities or persons from the same country.

India (IND) establishes their principal offshore relations with the British Virgin Islands, and it has been noted in studies that Indian entities related to tax havens, pay 30% less tax than another firms without these connections (Bryane and Goo, 2016). Indonesia (IDN) is also linked with the British Virgin Islands, with small relationships with another countries, such as Singapore and Samoa. Likewise, Indonesia is related, as an operation receiver, to Singapore and Thailand (THA).

Guernsey (GGY), a Crown dependency of the United Kingdom, is another international financial center which has larger operations with itself. There are other countries in Fig. 3, that contain minority operations compared to the rest of those analyzed. One of them is Venezuela (VEN), linked with the British Virgin Islands as well as Thailand, which is also connected with Indonesia. Russia (RUS), Cyprus (CYP), Switzerland (CHE) and the United Kingdom (GBR) have mainly small connections with themselves. The Cayman Islands (CYM) are linked as a receiver to Taiwan and to themselves, and the Bahamas (BHS) are connected with Panama and the British Virgin Islands. Again, the self-connections can be interpreted as the internal network to cover the offshoring operations of their external investors.

It is interesting to highlight that of the 22 countries that appear in the chord diagram, there are three that cover the greatest proportion of offshore relationships: the British Virgin Islands (VGB), Hong Kong, second, and Singapore. The high participation of the British Virgin Islands within the world of tax havens, being an area highly welcomed by countries of different regions as a financial center. This is in correspondence with the research developed by Garcia-Bernardo et al. (2017), where it is stated that the majority of shell entities are located in VGB. Thus, VGB constitutes a highly used tax haven due to the existing gaps in its tax and regulatory laws and the facilities it offers in the creation of offshore entities, which is reflected as VGB being the nation with the lowest score regarding regulatory gaps and secrecy by Corporate Tax Haven Index (Robertson, 2019). It is also noted that most of the countries studied have relations not only with other regions, but also with companies and entities from the same country.

### 4.3 Network structure for top countries

**Figure 4:**
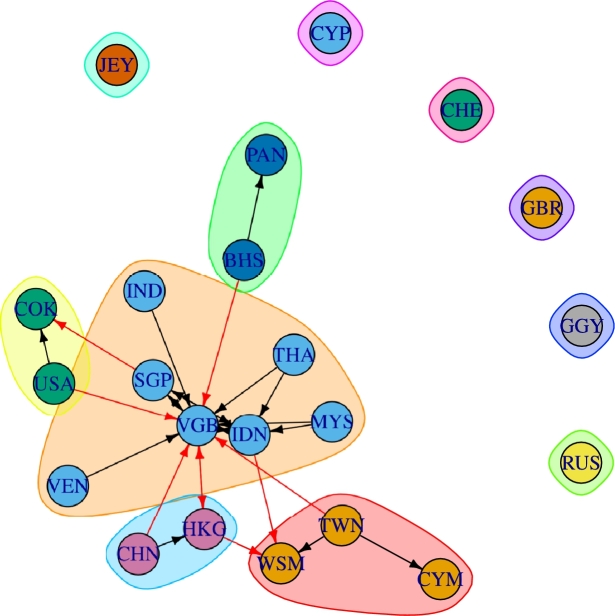
Network structure and clusters for top countries in the database. See acronyms of countries in Table 2.

Fig. 4 depicts the connectivity structure of the countries with a high number of occurrences in the database, corresponding to the top 0.1 percent of connections between countries, (quantile (q=0.999), $\theta_{q}=2.92$). According to $\theta_{q}=2.92$, the connected countries remaining are the 22 depicted, whether a country is present in the graph as an isolated node is due to a self-connection that exceeds the threshold. Then, the clusters that maximizes the modularity, which is a quality index for graph clustering, are found. The optimization is performed by maximizing the modularity measure over all possible partitions, via Integer Linear Programming (Brandes et al., 2008).

One can appreciate that the most interconnected tax haven is the British Virgin Islands (VGB), followed by Hong Kong (HKG), Indonesia (IDN), Singapore (SGP) and China (CHN). It can be observed that tax havens are moving to Asia, since the central (larger) cluster is composed mainly of Asian nodes (countries), with the British Virgin Islands (VGB) as a central hub of this cluster as well as for the whole network as shown in Fig. 4. China (CHN) and Hong Kong (HKG) are together in a cluster, where China is emitting transactions while Hong Kong is acting as a transmitter of offshoring operations to the largest cluster, as well to a cluster composed of Samoa (WSM), which is a predominant receiver of offshoring operations; Taiwan (TWN), an operations emitter; and the Cayman Islands (CYM), a receiver. Another cluster is created by the United States (USA), an emitter, and the Cook Islands (COK), a receiver. The Bahamas (BHS) act as an emitter connected to the main cluster and hub (VGB) and to the receiver node Panama (PAN). Finally, the disconnected nodes are the United Kingdom (GBR), Switzerland (CHE), Cyprus (CYP), Russia (RUS), Jersey (JER) and Guernsey (CGY). According to Kemme et al. (2017), this can be an indicator that although investors in general value close proximity, and similarities in legal systems and country governance, they prefer transactions and information privacy to avoid detection and persecution. Thus, when inter-country tax and investments information exchange agreements have been signed, people and companies could move their money to another destinations.

### 4.4 Offshore map relations to geographic regions

**Figure 5:**
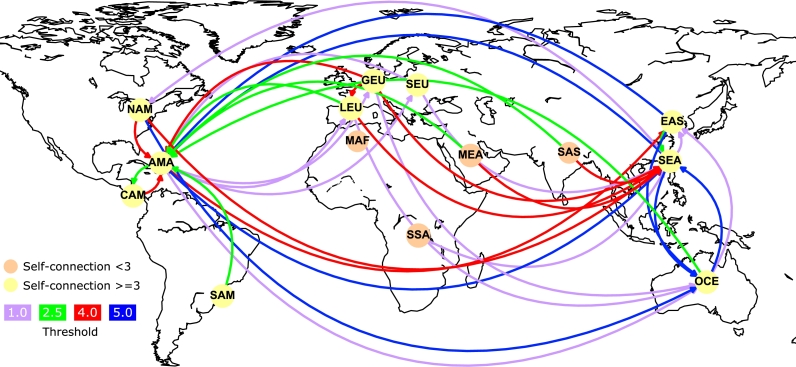
Geographic representation of Offshoring for worldwide regions. Regions' connectivity for different values of *θ* = {1.0,2.5,4.0,5.0} in purple, green, red, and blue links, respectively.

**Figure 6:**
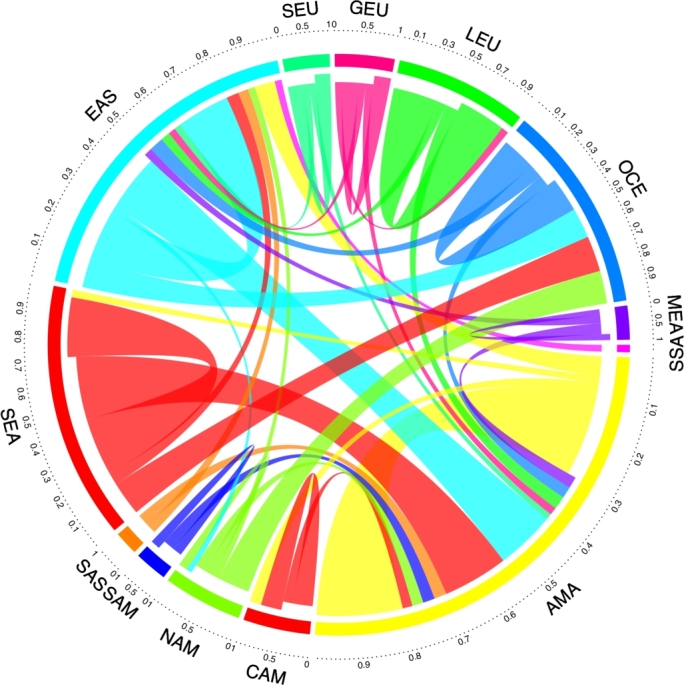
Offshoring map (chord-diagram) for world regions. 80th percentile of regions, quantile *q* = 0.8, trimming for weights below threshold of *θ*_*q*_ = 1.89. See acronyms of world regions in Table 3.

Fig. 5 depicts the geographic representation of the involved geographical regions and their relationships, according the Offshore Leaks database. The relationships between regions are represented for different values of the threshold θ={1,2.5,4,5} in purple, green, red, and blue arcs, respectively. The arc direction is given by the connections above the threshold. A node (region) with an outgoing arc accounts for a flow of outgoing operations that is larger than the threshold and a flow of incoming operations that is smaller than the threshold. A node (region) with an incoming arc accounts for the contrary. Strong self-connections (θ>=3) are represented as a yellow-filled circle and weak self-connections as bronze-filled circles (θ<3). These results are presented and discussed using the chord-diagram for a better visual representation of the relationships between regions in Fig. 6.

The principal involved regions in the offshoring network are listed with their acronyms in Table 3. These regions are the American Antilles, Central America, Eastern Asia, Germanic Europe, Latin Europe, Maghreb Africa, the Middle East, North America, Oceania, Sub-Saharan Africa, Slavic Europe, South America, South Asia and South East Asia.

**Table 3:** Regions' acronyms.

Region	Acronym
American Antilles	AMA
Central America	CAM
Eastern Asia	EAS
Germanic Europe	GEU
Latin Europe	LEU
Maghreb Africa	MAF
Middle East	MEA
North America	NAM
Oceania	OCE
Sub-Saharan Africa	SSA
Slavic Europe	SEU
South America	SAM
South Asia	SAS
South East Asia	SEA

Fig. 6 shows the offshoring network relationship structure for the connections' 80th percentile for the world geographical regions obtained from the mesoscopic configuration of the network defined in the previous section (Section 3). The size of the segments in the chord diagram is proportional to the number of countries in the geographical block and their weighted importance in terms of *R* (see Eq. (2)). The width of the links between segments indicates countries' connections that move from one region to another. Clearly, the links are thicker when they connect the largest blocks and according to the weighted relationship *R* between countries belonging the group. One should observe that the chord diagrams represent the outgoing influences as connections that have a larger gap from the departing region/country, and the incoming influences are represented by a smaller gap from the arriving region/country.

For example, the relationship between EAS (Eastern Asia) and AMA (American Antilles) indicates a strong relationship between entities belonging to the Eastern Asian (EAS) countries offshoring to the Antilles. Similarly, the relationship between South-East Asia (SEA) and the Antilles (AMA) indicates that the Antilles is receiving numerous offshoring operations from these regions (EAS, SEA). Likewise, East Asia has large offshore relationships with companies based in Oceania (OCE) and with other entities settled in the East Asia region. Analyzing South East Asia, their offshoring entities, in addition to being related to the Antilles, as mentioned previously, are also related to Oceania and companies from the region. In the case of Slavic Europe, a small number of the relationships are developed with entities from South East Asia and the Antilles, the prevailing offshoring relationships are inside this region. Germanic Europe, to a smaller scale, establishes offshore relations with South East Asia, the Antilles and Latin Europe. In Slavic Europe (SEU), Germanic Europe (GEU) and Latin Europe (LEU), their offshore entities predominantly establish relations with firms from the same region. On the other hand, the Antilles does not exert a large influence on other regions but has a extensive self-connection, which indicates a strong presence of relationships among the countries/entities inside the region.

Oceania is a region that mainly receives offshoring operations, approximately 70% in comparison to the emitted operations (30%). From this 70%, a smaller part corresponds to internal operations and the larger corresponds to external (countries) companies. This proportion means that most of the offshoring entities are from regions such as North America, South East Asia and Eastern Asia, with links to Oceania by relations based in address, intermediaries and officers, among others. On the other hand, the Middle East (MEA) received operations from their own entities, but their entities also establish relations with South East Asia and the Antilles.

Central America (CAM) establishes operations with their own entities and with the Antilles, in both directions, as a receiver and as a emitter. In North America (NAM), more than a half of the offshoring operations are between their entities and regions, such as Oceania, the Antilles and South Easts Asia, which means that North America is a region where large offshored entities are generated and where connections such as intermediaries, officers and address are established with firms or persons from the aforementioned receiving regions.

There are three remaining regions to be included in the analysis: South America (SAM), Sub-Saharan Africa (SSA) and South Asia (SAS), which are worth studying even though they have few relationships compared to the other analyzed regions. South America is linked basically with their own entities and with the Antilles; Sub-Saharan Africa is only linked with South East Asia; and South Asia is connected with the Antilles and South East Asia.

Up to this point, it is valid to highlight that from the thirteen regions involved, only four account for the major offshored operations: Antilles (the largest one), Eastern Asia, South East Asia and Oceania. Of all the studied regions, the predominant behavior is that these regions contain offshored entities that establish relationships with other companies or people from the same region and from other regions. Only Antilles and Oceania predominantly receive operations, based on connections with intermediaries, officers, and addresses, among others. The internal relationships can be seen as the internal network of entities to cover the traces of the receiving offshoring operations from other regions.

## 5 Conclusions

Using network modeling and analysis techniques, it was possible to characterize the connectivity structure for the top 22 countries in the ICIJ database and to identify the central actors and their influence in the offshoring network. The network was built from the different relations occurring between emitting and receiving entities in the ICIJ database and aggregated for their corresponding countries. The network of entities has been represented mesoscopically at the level of countries and geographical regions, and their intra- and inter-relationships have been mapped.

The findings provide a number of relevant conclusions. As commented before, we have identified the main offshoring regions/countries and how they are related to the rest of the worldwide actors. The most prominent regions identified as offshoring receptors are the American Antilles (AMA), South East Asia (SEA), Eastern Asia (EAS), and Oceania (OCE).

The most prevalent offshore receiving jurisdictions were identified as the British Virgin Islands (VGB), Hong Kong (HKG), Singapore (SGP), Indonesia (IDN), the Cook Islands (COK) and Samoa (WSM). The British Virgin Islands are the most predominant actor, serving as a hub in the offshoring network connecting countries from different regions.

The dimension of internal relationships is important, since it can give a measurement of the internal network used by the countries to hinder the tracking of money in tax havens. The first conclusion is that the offshoring size of regions and the number of offshoring countries has grown and diversified. A second conclusion is that these regions and countries have a different role and importance, as described in this work. Thus, the “traditional” tax havens in the Antilles and Central America continue to play a relevant role, however, Switzerland seems to have lost relevance. Although there is no evidence of the impact of tax information exchange agreements yet (Kemme et al., 2017), they could be one of factors that is changing the status quo. This assertion should be taken cautiously because more research is required. On the other hand, Asia has strongly emerged as a tax haven, and its evolution should be watched.

Building a network of offshoring entities and describing it on a mesoscopic scale, in this case by countries and geographical regions, offered interesting insights into the worldwide offshoring system. Applying this approach in order to tackle a variety of socio-economic problems (González et al., 2018a, González et al., 2018b) is of interest to obtain a plausible description of the structure and intra/inter-relationships of the system components. Using the knowledge of existing connections, regulatory entities, both nationally and internationally, can act in these networks with the aim of increasing their control and eliminating fraudulent behavior. The origin of tax havens was based on the search for capital investments, this initial origin has tended to deviate from its purpose to hide transfer and concealment of illicit capital. In this sense, the present investigation is a valid study that allows to discover structures of the offshore relations between regions and countries around the world, to better regulate such activities.

The selected level of detail for this work (country level), might be a limitation in terms of the patterns that were discovered. As a further project, we can apply network analysis techniques as well as a complex network characterization to the whole offshoring network at microscopic level (i.e. enterprise information) to determine relationships that can be linked not only to the geographical analysis conducted in this work but also to other types of structures and behavior that may emerge in the offshoring network.

## Declarations

### Author contribution statement

M. Gonzalez: Performed the experiments; Analyzed and interpreted the data; Wrote the paper.

D. Dominguez: Conceived and designed the experiments; Analyzed and interpreted the data; Wrote the paper.

O. Pantoja: Analyzed and interpreted the data; Wrote the paper.

P. Pico: Performed the experiments.

M. Mateos: Conceived and designed the experiments; Performed the experiments.

M. Alonso: Analyzed and interpreted the data; Wrote the paper.

### Funding statement

This work was funded by UDLA-SIS.MGR.20.01

### Competing interest statement

The authors declare no conflict of interest.

### Additional information

No additional information is available for this paper.
